# TGF-β-Activated Kinase 1 (TAK1) Signaling Regulates TGF-β-Induced WNT-5A Expression in Airway Smooth Muscle Cells via Sp1 and β-Catenin

**DOI:** 10.1371/journal.pone.0094801

**Published:** 2014-04-11

**Authors:** Kuldeep Kumawat, Mark H. Menzen, Ralph M. Slegtenhorst, Andrew J. Halayko, Martina Schmidt, Reinoud Gosens

**Affiliations:** 1 Department of Molecular Pharmacology, University of Groningen, Groningen, the Netherlands; 2 Groningen Research Institute for Asthma and COPD, University of Groningen, Groningen, the Netherlands; 3 Departments of Physiology & Internal Medicine, University of Manitoba, Winnipeg, Canada; Temple University School of Medicine, United States of America

## Abstract

WNT-5A, a key player in embryonic development and post-natal homeostasis, has been associated with a myriad of pathological conditions including malignant, fibroproliferative and inflammatory disorders. Previously, we have identified WNT-5A as a transcriptional target of TGF-β in airway smooth muscle cells and demonstrated its function as a mediator of airway remodeling. Here, we investigated the molecular mechanisms underlying TGF-β-induced WNT-5A expression. We show that TGF-β-activated kinase 1 (TAK1) is a critical mediator of WNT-5A expression as its pharmacological inhibition or siRNA-mediated silencing reduced TGF-β induction of WNT-5A. Furthermore, we show that TAK1 engages p38 and c-Jun N-terminal kinase (JNK) signaling which redundantly participates in WNT-5A induction as only simultaneous, but not individual, inhibition of p38 and JNK suppressed TGF-β-induced WNT-5A expression. Remarkably, we demonstrate a central role of β-catenin in TGF-β-induced WNT-5A expression. Regulated by TAK1, β-catenin is required for WNT-5A induction as its silencing repressed WNT-5A expression whereas a constitutively active mutant augmented basal WNT-5A abundance. Furthermore, we identify Sp1 as the transcription factor for WNT-5A and demonstrate its interaction with β-catenin. We discover that Sp1 is recruited to the WNT-5A promoter in a TGF-β-induced and TAK1-regulated manner. Collectively, our findings describe a TAK1-dependent, β-catenin- and Sp1-mediated signaling cascade activated downstream of TGF-β which regulates WNT-5A induction.

## Introduction

WNT-5A is a member of the Wingless/integrase 1 (WNT) family of secreted glycoproteins. There are 19 WNT ligands known in humans that act through 10 Frizzled (FZD) receptors, low-density lipoprotein receptor-related protein (LRP) 5/6 co-receptors and many non-FZD receptors, including ROR1, ROR2, RYK [1]. WNT signaling is broadly subdivided into two main streams- canonical (β-catenin-dependent) and non-canonical (β-catenin-independent) WNT signaling. In the canonical signaling, binding of a WNT ligand to a FZD receptor and LRP5/6 co-receptors activates signaling mechanisms resulting in stabilization of the transcriptional co-activator β-catenin, leading to its accumulation in the cytosol. Stabilized β-catenin translocates to the nucleus where it partners with the T-cell factor/lymphoid enhancer-binding factor (TCF/LEF) transcription factors and activates target gene transcription. Non-canonical WNT signaling functions exclusive of β-catenin and LRP5/6 and involves a multitude of pathways regulating gene transcription, cytoskeletal reorganization, cell polarity and cell movements. WNT/Ca^2+^ and WNT/planar cell polarity (PCP) are the best characterized non-canonical WNT signaling pathways among others. In the WNT/Ca^2+^ signaling, binding of WNT ligands to FZD or non-FZD receptors activates calcium-dependent signaling molecules, including protein kinase C (PKC), Ca^2+^/calmodulin-dependent protein kinase II (CaMKII) and nuclear factor of activated T-cell (NFAT), whereas the WNT/PCP pathway involves activation of the RhoA signaling or c-Jun N-terminal Kinases (JNKs) via small Rho-GTPases [1].

WNT-5A is a crucial signaling molecule which primarily acts through non-canonical WNT signaling and plays key roles in embryonic development and post-natal homeostatic processes [2], [3]. It is involved in lung [4], heart [5] and mammary gland morphogenesis [6] and regulates stem cell renewal and tissue regeneration [7], [8]. In parallel, WNT-5A has been linked to inflammation [9] and various malignancies [10].

Furthermore, WNT-5A has been very closely associated with fibrosis. Increased amount of WNT-5A is reported in lung fibroblasts of pulmonary fibrosis patients where it regulates proliferation and promotes cell viability [11]. Similarly, studies have implicated WNT-5A expression and signaling in renal [12] and hepatic [13] fibrosis. WNT-5A signaling has also been implicated in ciliopathies [14] and WNT-5A antagonism has been shown to counteract vascular calcification [15].

We have recently reported increased WNT-5A expression in asthmatic airway smooth muscle cells [16]. We have shown that TGF-β induces WNT-5A expression in airway smooth muscle cells where it mediates the expression of extracellular matrix proteins (ECM) [16]. TGF-β also induces WNT-5A expression in pancreatic cancer cells [17]. Similarly, the pro-inflammatory cytokines-IL-1β [18], TNF-α [19], LPS/IFNγ [20], IL-6 family members- leukemia inhibitory factor (LIF) and cardiotrophin-1 (CTF-1) [21] and high extracellular Ca^2+^ concentration [22] have also been shown to augment WNT-5A expression in various cell types.

While our knowledge about the involvement of WNT-5A in various physiological and pathological processes is evolving rapidly along with the identification of novel inducers, the understanding of mechanisms regulating WNT-5A expression and homeostasis remains poor. In this study, we have investigated the molecular mechanisms involved in TGF-β-induced WNT-5A expression using airway smooth muscle cells as model system.

TGF-β is a pleiotropic cytokine with functions as diverse as embryonic development and maintenance of adult tissue homeostasis to regulating stem cell renewal, cell fate determination and cellular proliferation [23], [24]. Binding of TGF-β to its receptors leads to phosphorylation and dimerization of SMAD2/3 and generation of a heterotrimeric complex with SMAD4 which translocates to the nucleus and activates TGF-β-responsive genes. Besides, TGF-β can signal in a SMAD-independent manner through activation of TGF-β-activated kinase 1 (TAK1), p38, extracellular signal-regulated kinases 1/2 (ERK1/2), JNK, phosphatidylinositol 3-kinase (PI3K)/AKT, small Rho-GTPases and Nuclear Factor κB (NFκB) to name a few [25].

TAK1, first identified as a mitogen-activated kinase kinase kinase (MAP3K) activated by TGF-β, is a critical regulator in inflammatory, immune and stress response signaling [26], [27]. TAK1 constitutes an integral part of pro-inflammatory cytokine signaling, activating NFκB and MAPK pathways [27]. Besides, TAK1 also mediates the SMAD-independent arm of the TGF-β signaling pathway and regulates various TGF-β-induced cellular responses [27], [28].

Here, we investigated the molecular mechanisms involved in TGF-β-induced WNT-5A expression using airway smooth muscle cells as 1] airway smooth muscle cells are key structural and functional component of airways and major contributor of airway remodeling in asthma and 2] TGF-β upregulates WNT-5A expression in these cells. We examined the participation of various TGF-β-activated pathways and demonstrate that TAK1 via p38 and JNK mediates WNT-5A expression. Further, we determined an unanticipated role for β-catenin in WNT-5A expression and describe its regulation by TAK1. Finally, we identify Sp1 as the transcription factor for *WNT-5A* and demonstrate a link between TAK1, β-catenin and Sp1.

## Materials and Methods

### Reagents

Recombinant human TGF-β_1_ and rat anti-WNT-5A antibody were from R&D systems (Abingdon, UK). siRNAs specific for human TAK1, human CUTL1, human TCF4 and human ETS1, rabbit anti-Sp1 (PEP2) X TransCruz, mouse anti-GAPDH, mouse anti-β-actin, horseradish peroxidase (HRP)-conjugated chicken anti-rat antibody and Protein A-agarose were purchased from Santa Cruz Biotechnology (Santa Cruz, CA, USA). Rabbit anti-phospho-Thr183/Tyr185-SAPK/JNK antibody and rabbit anti-phospho-Thr180/Tyr182-p38 MAPK (D3F9) antibody were obtained from Cell Signaling Technology (Beverly, MA, USA). Mouse anti-total β-catenin antibody was from BD Biosciences (San Jose, CA, USA) and mouse anti-active β-catenin antibody (clone 8E7) was obtained from Millipore (Amsterdam, the Netherlands). Cycloheximide, IGEPAL CA-630, HRP-conjugated goat anti-mouse antibody and HRP-conjugated goat anti-rabbit antibody were obtained from Sigma (St. Louis, MO, USA). Human β-catenin and non-targeting siRNA were procured from Qiagen (Venlo, the Netherlands). X-tremeGENE siRNA and X-tremeGENE DNA HP transfection reagents were purchased from Roche Applied Science (Mannheim, Germany). LL-Z1640-2 was obtained from Bioaustralis (Smithfield, NSW, Australia). Y-27632 dihydrochloride, LY294002 hydrochloride, SB203580, SP600125 and Mithramycin A were from Tocris (Bristol, UK) and SIS3 and Bisindolylmaleimide I (BIM) were purchased from Calbiochem (La Jolla, CA, USA). All other chemicals were of analytical grade.

### Cell culture

Three human airway smooth muscle cell lines, immortalized by human telomerase reverse transcriptase (hTERT) [29] were used for all the experiments. The primary cultured human airway smooth muscle cells used to generate each hTERT immortalized cell line were prepared as described previously [29]. All procedures were approved by the Human Research Ethics Board (University of Manitoba). hTERT-airway smooth muscle cell lines were maintained on uncoated plastic dishes in Dulbecco's modified Eagle's medium (DMEM) supplemented with antibiotics (50 U/ml streptomycin, 50 µg/ml penicillin) and 10% (v/v) fetal bovine serum (FBS). For each experiment, hTERT-airway smooth muscle cell lines (airway smooth muscle cells) derived from two to three different donors were used for repeated measurements. Cells were serum-deprived in DMEM supplemented with antibiotics and ITS (5 µg/ml insulin, 5 µg/ml transferrin, and 5 ng/ml selenium) before each experiment. When applied, inhibitors were added 30 min before the TGF-β stimulation.

### siRNA transfection

Airway smooth muscle cells were grown to ∼90% confluence in 6-well cluster plates and transfected with 200 pmol of specific siRNA in serum and antibiotic free DMEM with X-tremeGENE siRNA transfection reagent. Control transfections were performed using a non-targeting control siRNA. After 6 hours of transfection, medium was replaced with DMEM supplemented with antibiotics and ITS for a period of 42 hours before TGF-β stimulation.

### S33Y-β-catenin DNA transfection

Airway smooth muscle cells grown to ∼90% confluence in 6-well cluster plates were transfected with 1 µg of mutant S33Y-β-catenin plasmid (AddGene plasmid 19286, AddGene public repository, Cambridge, MA, USA) [30] in serum and antibiotic free DMEM using X-tremeGENE HP DNA transfection reagent. 2 µg of Green Fluorescent Protein (GFP) expression vector was transfected as control. After 6 hours of transfection, medium was replaced with DMEM supplemented with antibiotics and 10% (v/v) fetal bovine serum (FBS) for 18 hours. Cells were then serum-deprived in DMEM supplemented with antibiotics and ITS for 24 hours before the TGF-β stimulation.

### RNA isolation and real-time PCR

Total RNA was extracted using the Nucleospin RNAII kit (Macherey-Nagel, Duren, Germany) as per the manufacturer's instructions. Equal amounts of total RNA were then reverse transcribed using the Reverse Transcription System (Promega, Madison, USA). 1 µl of 1∶2 diluted cDNA was subjected to real-time PCR, which was performed with the Illumina Eco Personal QPCR System (Westburg, Leusden, the Netherlands) using FastStart Universal SYBR Green Master (Rox) from Roche Applied Science (Mannheim, Germany). Real time PCR was performed with denaturation at 94°C for 30 seconds, annealing at 59°C for 30 seconds and extension at 72°C for 30 seconds for 40 cycles followed by 10 minutes at 72°C. Real time PCR data was analyzed using the comparative cycle threshold (C_q_: amplification cycle number) method. The amount of target gene was normalized to the endogenous reference gene 18S ribosomal RNA (ΔC_q_). Relative differences were determined using the equation 2^(−ΔΔCq)^. Primers used to analyze gene expression are: WNT-5A Fwd 5′- GGGTGGGAACCAAGAAAAAT -3′ and Rev 5′- TGGAACCTACCCATCCCATA -3′; TAK1 Fwd 5′- CTTGGATGGCACCTGAAG -3′ and Rev 5′- CAGGCTCTCAATGGGCTTAG -3′; Collagen IαI Fwd 5′- AGCCAGCAGATCGAGAACAT -3′ and Rev 5′- TCTTGTCCTTGGGGTTCTTG -3′; Fibronectin Fwd 5′- TCGAGGAGGAAATTCCAATG -3′ and Rev 5′- ACACACGTGCACCTCATCAT -3′; β-catenin Fwd 5′- CCCACTAATGTCCAGCGTTT -3′and Rev 5′- AATCCACTGGTGAACCAAGC -3′; CUTL1 Fwd 5′- GCTGTTGCTGGAGAAGAACC -3′and Rev 5′- GGTCTTTCCCTTTCCTCCTG -3′; TCF4 Fwd 5′-CGTAGACCCCAAAACAGGAA -3′and Rev 5′- TCCTGTCGTGATTGGGTACA -3′; ETS1 Fwd 5′- CCAATCCAGCTATGGCAGTT -3′and Rev 5′- TTCCTCTTTCCCCATCTCCT -3′; Sp1 Fwd 5′- GGAGAGCAAAACCAGCAGAC -3′ and Rev 5′- AAGGTGATTGTTTGGGCTTG -3′ and 18S rRNA Fwd 5′- CGCCGCTAGAGGTGAAATTC -3′and Rev 5′- TTGGCAAATGCTTTCGCTC -3′.

### *In silico* promoter analysis

*WNT-5A* promoter sequences for both the alternative promoters A and B were derived from human chromosome 3 genome (NCBI accession # NT_022517) in consultation with earlier reports [31]–[33]. Sequences were screened to identify the putative transcription factor binding sites using online program PROMO version 3 [34], [35]. The parameters were set to detect only human transcription factor binding sites with maximum matrix dissimilarity rate set at 5%.

### Chromatin immunoprecipitation (ChIP) assay

ChIP analysis was performed using the SimpleChIP Enzymatic Chromatin IP Kit (Agarose Beads) from Cell Signaling Technology (Beverly, MA, USA) as per manufacturer's instructions. Briefly, 1×10^7^ airway smooth muscle cells were fixed in formaldehyde to final concentration of 1% for 10 minutes and then stopped by adding glycine. Cross-linked chromatin was digested using Micrococcal nuclease at 37°C for 20 minutes followed by a brief sonication to generate 200–500 bp DNA fragments. Sheared chromatin was incubated with anti-Sp1 (PEP2) X TransCruz reagent (Santa Cruz Biotechnology, Santa Cruz, CA, USA) or normal rabbit antibody (IgG) as negative control and precipitated using Protein G-agarose beads. Immunoprecipitated chromatin complexes were washed sequentially in Low- and High- salt wash buffers and protein-DNA cross-links were reversed in presence of Proteinase K at 65°C for 4 hours. DNA fragments were purified using the spin columns supplied in the kit as per recommendations. 2 µl of DNA from each sample was used as a template for PCR amplification. PCR was performed with denaturation at 94°C for 30 seconds, annealing at 59°C for 30 seconds and extension at 72°C for 30 seconds for 40 cycles followed by 10 minutes at 72°C using primers designed to amplify the region encompassing putative Sp1 binding sites on *WNT-5A* promoter A Fwd 5′- ACAGGATCGCGTGGAAATCT -3′and Rev 5′- GAAGCTGCCCACCTCCTC -3′.

### L-cell conditioned medium preparation

Control and WNT-3A conditioned medium from L-cells were prepared as described previously [16].

### Preparation of cell lysates

The whole cell extracts were either prepared as described previously [16] using SDS lysis buffer or by direct lysis in 2X Laemmli loading buffer.

### Co-Immunoprecipitation

For co-immunoprecipitation assay, airway smooth muscle cells were washed twice with ice-cold PBS and lysed in 1% IGEPAL buffer (20 mM Tris-HCl pH7.5, 120 mM NaCl, 1% IGEPAL CA-630, 2 mM EDTA, 1 mM EGTA, 10 µg/ml Leupeptin, Aprotinin and Pepstatin, 1 mM NaF, 1 mM Na_3_VO_4_, 1 mM PMSF and 1 mM β-glycerophosphate) on ice, scraped and collected in a microfuge. The collected lysate was further incubated at 4°C with constant rotation for 4 hours. Lysates were then cleared by centrifugation at 18000 *g* for 10 min at 4°C and supernatant was collected. Protein concentrations were measured using the BCA assay (Pierce) and 500 µg of protein lysate was incubated with 2 µg anti-Sp1 antibody overnight at 4°C. Immunocomplexes were then incubated with 30 µl of Protein A-agarose slurry for 4 hours with constant rotation at 4°C. Protein A-agarose-bound immunocomplexes were precipitated by centrifugation at 4000 *g* for 5 min at 4°C and washed three times with lysis buffer. Finally, 2X Laemmli buffer was added to the precipitates and heated for 5 min at 95°C. The heated lysates were cleared by centrifugation at 4000 *g* for 5 min, supernatant collected and stored at −20°C until further use.

### Western analysis

Protein samples were subjected to electrophoresis, transferred to nitrocellulose membranes, and analyzed for the proteins of interest using specific primary and HRP-conjugated secondary antibodies. Bands were subsequently visualized using the G-box gel documentation system (Syngene, Cambridge, UK) using enhanced chemiluminescence reagents and were quantified by densitometry using Genetools software.

### Data Analysis

Values reported for all data are represented as mean ± SEM. The statistical significance of differences between means was determined on log transformed data by Student's *t*-test, by 1-way ANOVA or by 2-way ANOVA, followed by Student-Newman Keuls or Bonferroni multiple comparisons test, where appropriate. Differences were considered to be statistically significant when p<0.05.

## Results

### TAK1 mediates TGF-β-induced WNT-5A expression

TGF-β activates multiple pathways, both SMAD-dependent and -independent, downstream of its receptor. We targeted key pathways to identify the signaling cascades involved in WNT-5A expression by TGF-β in airway smooth muscle cells. We observed that pharmacological inhibition of SMAD3 (SIS3; 3 µM), Rho-associated protein kinase (ROCK) (Y27632; 1 µM), PI3K (LY294002; 3 µM), glycogen synthase kinase (GSK) -3 (SB216763; 10 µM) and PKC (BIM; 3 µM), failed to reduce WNT-5A induction by TGF-β (Figure S1A-E). Surprisingly, the SMAD3 inhibitor SIS3 significantly increased WNT-5A mRNA abundance by ∼2-fold in comparison to both the basal and TGF-β-stimulated conditions (Figure S1A) whereas GSK-3 inhibition by SB216763 also lead to a modest but significant increase in WNT-5A induction at the basal level (fold-induction 1.7±0.4) (Figure S1D).

**Figure 1:**
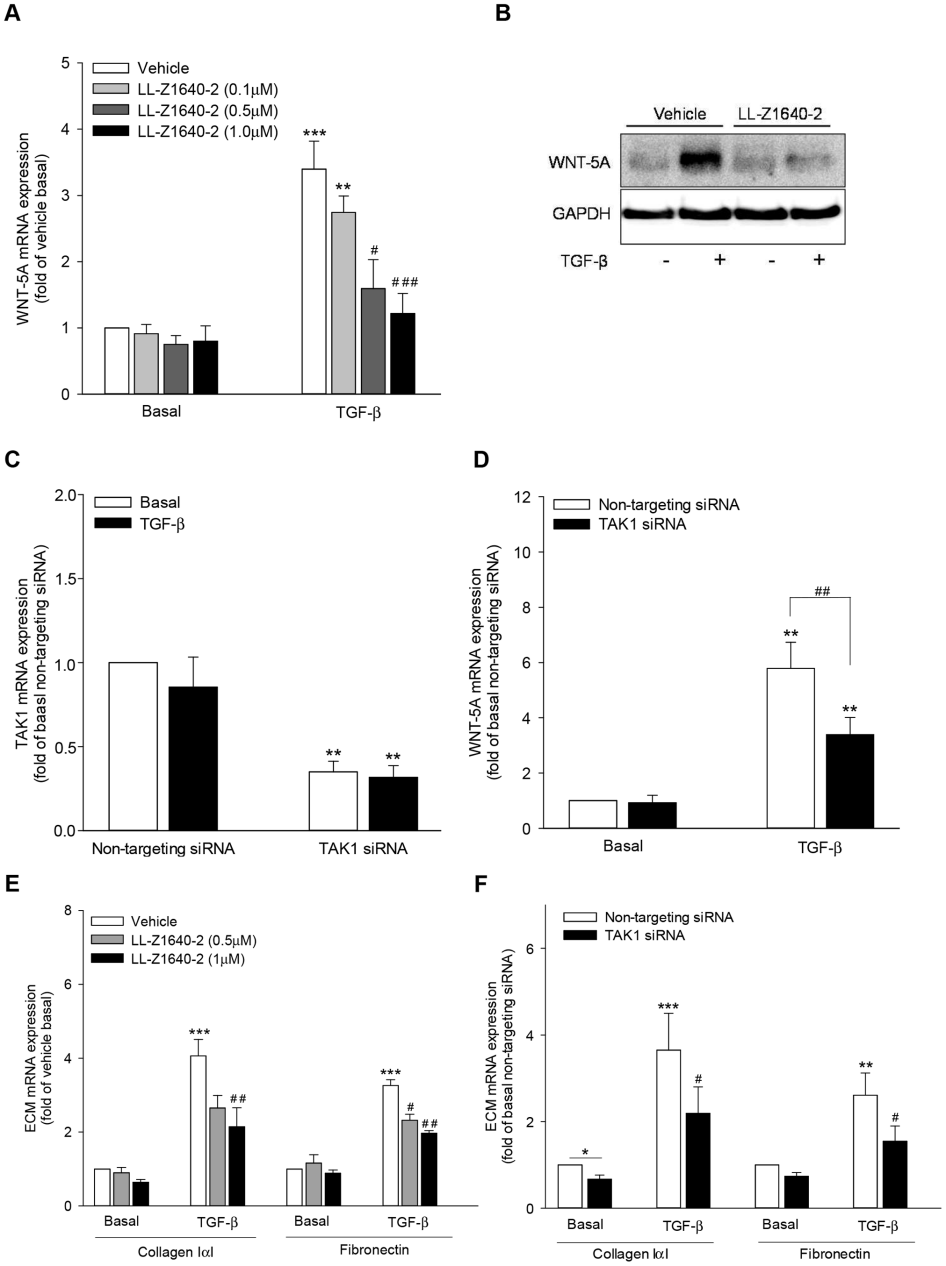
TAK1 regulates TGF-β-mediated WNT-5A induction in airway smooth muscle cells. (A, E) Airway smooth muscle cells were either left unstimulated (vehicle basal) or stimulated with TGF-β (2 ng/ml) in the presence or absence of LL-Z1640-2 (0.1 µM, 0.5 µM, 1.0 µM) for 24 hours. Expression of WNT-5A mRNA(A) and collagen IαI and fibronectin mRNA (E) was determined by qRT-PCR, corrected for 18S rRNA and expressed relative to vehicle basal. Data represent mean ± SEM of 4-5 independent experiments. **p<0.01, ***p<0.001 compared to vehicle basal, # p<0.05, ## p<0.01, ### p<0.001 compared to TGF-β-stimulated cells; 2-way ANOVA followed by Bonferroni multiple comparisons test. (B) Airway smooth muscle cells were stimulated with TGF-β (2 ng/ml) in the presence or absence of LL-Z1640-2 (0.5 µM) for 48 hours. Western analysis was performed on whole cells extracts for WNT-5A protein. Expression of GAPDH was analyzed as loading control. (C–D, F) Airway smooth muscle cells were transfected with TAK1-specific siRNA or a non-targeting siRNA as control. Subsequently, cells were stimulated with TGF-β (2 ng/ml) for 24 hours and analyzed for the expression of TAK1 mRNA (C), WNT-5A mRNA (D) and collagen IαI and fibronectin mRNA (F) by qRT-PCR and expressed relative to non-targeting siRNA-transfected, untreated control. Data represent mean ± SEM of 4 independent experiments. *p<0.05, **p<0.01, ***p<0.001 compared to non-targeting siRNA-transfected untreated control, #p<0.05, ## p<0.01 compared to non-targeting siRNA-transfected, TGF-β-stimulated cells; 1-way ANOVA followed by Newman-Keuls multiple comparisons test.

Notably, inhibition of TAK1 by LL-Z1640-2 attenuated WNT-5A mRNA expression in a dose-dependent manner with significant reduction at 0.5 µM and 1 µM by ∼75% and ∼91%, respectively (Figure 1A). Consistent with the mRNA data, TAK1 inhibition also abrogated the TGF-β-induced increase in WNT-5A protein expression (Figure 1B).

To further validate the role of TAK1 in WNT-5A induction, we employed TAK1-specific siRNA. Transfection of airway smooth muscle cells with TAK1 siRNA significantly repressed TAK1 transcripts to ∼30% of the baseline expression in both the unstimulated and TGF-β-stimulated airway smooth muscle cells in comparison to non-targeting siRNA transfected cells (Figure 1C). In agreement with the findings above using LL-Z1640-2, TAK1-specific siRNA significantly attenuated TGF-β-induced increase in abundance of WNT-5A transcripts by ∼50% (Figure 1D).

We have previously reported a role for WNT-5A in TGF-β-induced ECM production [16]. In line with that, both the inhibition and knock-down of TAK1 reduced TGF-β-induced ECM production, further confirming an upstream role for TAK1 in WNT-5A expression (Figure 1E, F).

Collectively, our data suggest that TAK1 specifically mediates TGF-β-induced WNT-5A production.

### TAK1-activated p38 and JNK signaling mediate TGF-β-induced WNT-5A expression

**Figure 2:**
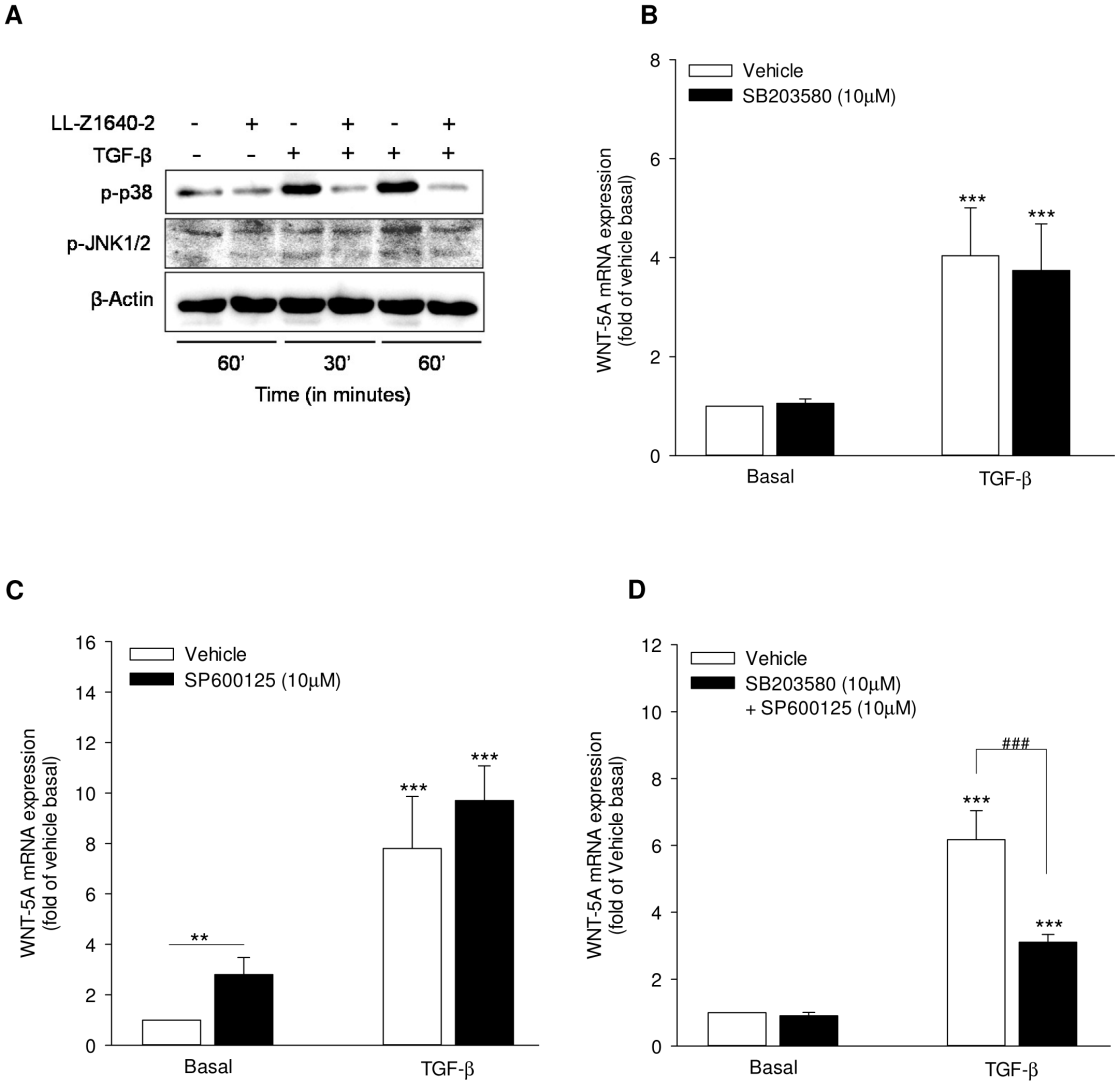
TAK1-activated p38/JNK signaling regulates WNT-5A induction in airway smooth muscle cells. (A) TAK1 activates p38 and JNK. Airway smooth muscle cells were stimulated with TGF-β (2 ng/ml) in the presence or absence of LL-Z1640-2 (0.5 µM) for 30 and 60 minutes. Whole cells extracts were immunoblotted for phospho-p38 and phospho-JNK using specific antibodies. Equal protein loading was verified by the analysis of β-actin. (B–D) p38 and JNK involvement in WNT-5A expression. Airway smooth muscle cells were stimulated with TGF-β (2 ng/ml) in the presence or absence of SB203580 (10 µM) or SP600125 (10 µM) or combination of both SB203580 and SP600125 (10 µM each) for 24 hours. RNA was isolated and WNT-5A mRNA expression was determined by qRT-PCR, corrected for 18S rRNA and expressed relative to vehicle basal. Data represent mean ± SEM of 4–6 independent experiments. **p<0.01, ***p<0.001 compared to vehicle basal, ### p<0.001 compared to TGF-β-stimulated cells; 1-way ANOVA followed by Newman-Keuls multiple comparisons test.

Next, we investigated the signaling mechanisms downstream of TAK1 activation which could be involved in WNT-5A induction by TGF-β in airway smooth muscle cells. TAK1 activates JNK and p38 pathways in multiple systems [27] which we sought to confirm in airway smooth muscle cells. We found that TGF-β induced activation of p38 and JNK, as indicated by their increased phosphorylation status, which was attenuated in the presence of the TAK1 inhibitor LL-Z1640-2 (Figure 2A). Next, we directly targeted p38 and JNK kinases to assess their effect on WNT-5A induction. Surprisingly, individual targeting of p38 (SB203580; 10 µM) and JNK (SP600125; 10 µM) by specific pharmacological inhibitors failed to lower TGF-β-induced WNT-5A mRNA expression (Figure 2B, C), whereas targeting p38 and JNK signaling simultaneously lead to significant attenuation of TGF-β-induced WNT-5A mRNA expression by ∼60% (Figure 2D).

Our data therefore suggest that TAK1 mediates TGF-β-induced p38 and JNK kinases activation which can redundantly mediate the downstream effects of TAK1 on WNT-5A induction.

### β-Catenin is involved in TGF-β-induced WNT-5A expression

**Figure 3:**
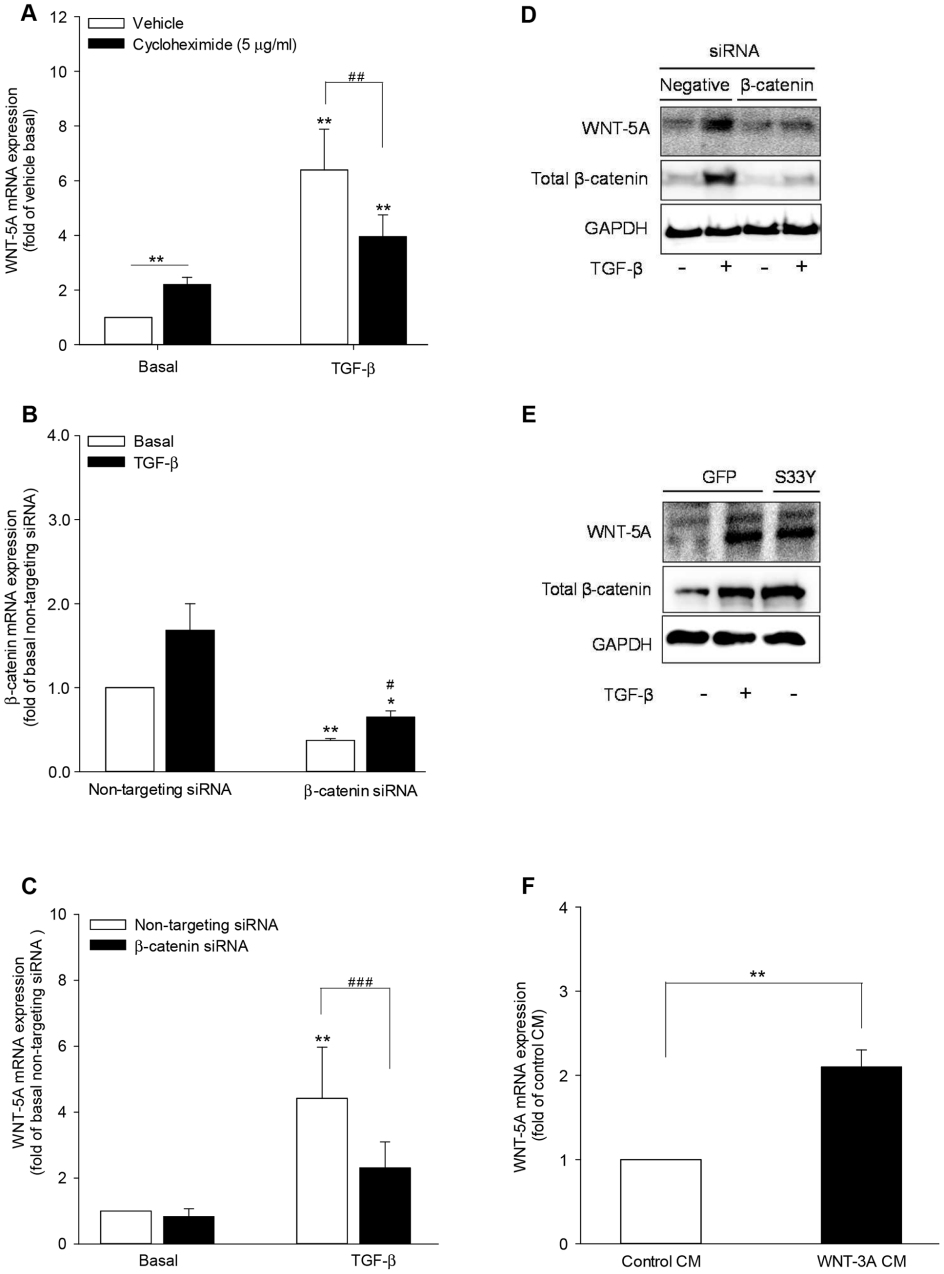
β-Catenin mediates TGF-β-induced WNT-5A expression in airway smooth muscle cells. (A) *De novo* protein synthesis is required for TGF-β-induced WNT-5A expression. Airway smooth muscle cells were either left unstimulated (vehicle basal) or stimulated with TGF-β (2 ng/ml) in the presence or absence of the protein synthesis inhibitor cycloheximide (5 µg/ml) for 24 hours. WNT-5A mRNA induction was evaluated by qRT-PCR. Data represent mean ± SEM of 4 independent experiments. **p<0.01, ***p<0.001 compared to vehicle basal, ## p<0.01 compared to TGF-β-stimulated cells; 2-tailed Student's *t* test for paired observations. (B-D) β-Catenin silencing reduces TGF-β-induced WNT-5A expression. Airway smooth muscle cells were transfected with β-catenin-specific siRNA or a non-targeting siRNA as control. Subsequently, cells were stimulated with TGF-β (2 ng/ml) for 24 hours (mRNA; B,C) or 48 hours (protein; D). (B,C) Expression of β-catenin mRNA (B) and WNT-5A mRNA (C) was determined by qRT-PCR and expressed relative to non-targeting siRNA transfected, untreated control. Data represent mean ± SEM of 5 independent experiments. *p<0.05, **p<0.01 compared to non-targeting siRNA-transfected, untreated control, # p<0.05, ### p<0.001 compared to non-targeting siRNA-transfected, TGF-β-stimulated cells; 2-tailed Student's *t* test for paired observations. (D) Western blot analysis was performed to analyze WNT-5A and β-catenin protein expression in whole cell extracts. Equal protein loading was verified by the analysis of GAPDH. (E) Forced increase in β-catenin abundance elevates WNT-5A protein level. Cells were transfected with S33Y-β-catenin mutant or a GFP expression vector as control. Subsequently, cells were either left untreated or stimulated with TGF-β (2 ng/ml) for 48 hours. Western blot analysis was performed to determine the abundance of WNT-5A and total β-catenin at protein level. GAPDH expression assessed as loading control. (F) Canonical WNT ligand stimulation increases WNT-5A gene expression. Cells were stimulated with L-cells-derived WNT-3A conditioned medium or control conditioned medium for 24 hours. Expression of WNT-5A mRNA was evaluated by qRT-PCR and expressed relative to control conditioned medium. Data represent mean ± SEM of 5 independent experiments. **p<0.01 compared to control conditioned medium; 2-tailed Student's *t* test for paired observations.

In order to further clarify the molecular mechanisms mediating WNT-5A induction, we targeted protein translation to address whether *de novo* protein synthesis is involved in TGF-β-induced WNT-5A expression in airway smooth muscle cells. Interestingly, while the presence of cycloheximide increased basal WNT-5A mRNA abundance (fold-induction 2.2±0.26); it significantly attenuated TGF-β-induced augmentation in WNT-5A transcript levels by ∼44% (Figure 3A).

We have earlier shown that TGF-β stabilizes the canonical WNT signaling effector and transcriptional co-activator β-catenin in airway smooth muscle cells which is affected by inhibition of *de novo* protein synthesis [36]; therefore we investigated the involvement of β-catenin in WNT-5A induction. Transfection of airway smooth muscle cells with β-catenin-specific siRNA significantly decreased the abundance of β-catenin transcripts in both the unstimulated and TGF-β stimulated cells confirming an effective knock-down (Figure 3B). Accordingly, β-catenin siRNA attenuated TGF-β-induced WNT-5A mRNA expression by ∼62% in comparison to non-targeted siRNA-transfected cells (Figure 3C). In accordance with the mRNA data, β-catenin knock-down abrogated TGF-β-induced WNT-5A expression at protein level as well (Figure 3D).

To further corroborate the role of β-catenin, we utilized degradation-resistant constitutively active β-catenin mutant (S33Y-β-catenin). This S33Y-β-catenin mutant has a serine to tyrosine substitution at amino acid position 33 rendering it unphosphorylatable by GSK-3 and therefore resistant to proteasomal degradation. Transfection of airway smooth muscle cells with S33Y-β-catenin lead to enhanced expression of total β-catenin in the cell (Figure 3E). Interestingly, this was sufficient to increase WNT-5A protein in the absence of TGF-β, remarkably similar to the level of WNT-5A in control vector-transfected TGF-β-treated cells (Figure 3E).

As β-catenin stabilization is a hallmark of canonical WNT signaling activation, we hypothesized that canonical WNT signaling can also increase WNT-5A expression. To test this hypothesis, we stimulated airway smooth muscle cells with WNT-3A conditioned medium. Remarkably, WNT-3A conditioned medium led to a 2-fold induction in WNT-5A transcript levels in airway smooth muscle cells when compared to control conditioned medium (Figure 3F).

Our data therefore suggest a central role for β-catenin in WNT-5A induction.

### TAK1 signaling regulates β-catenin

**Figure 4:**
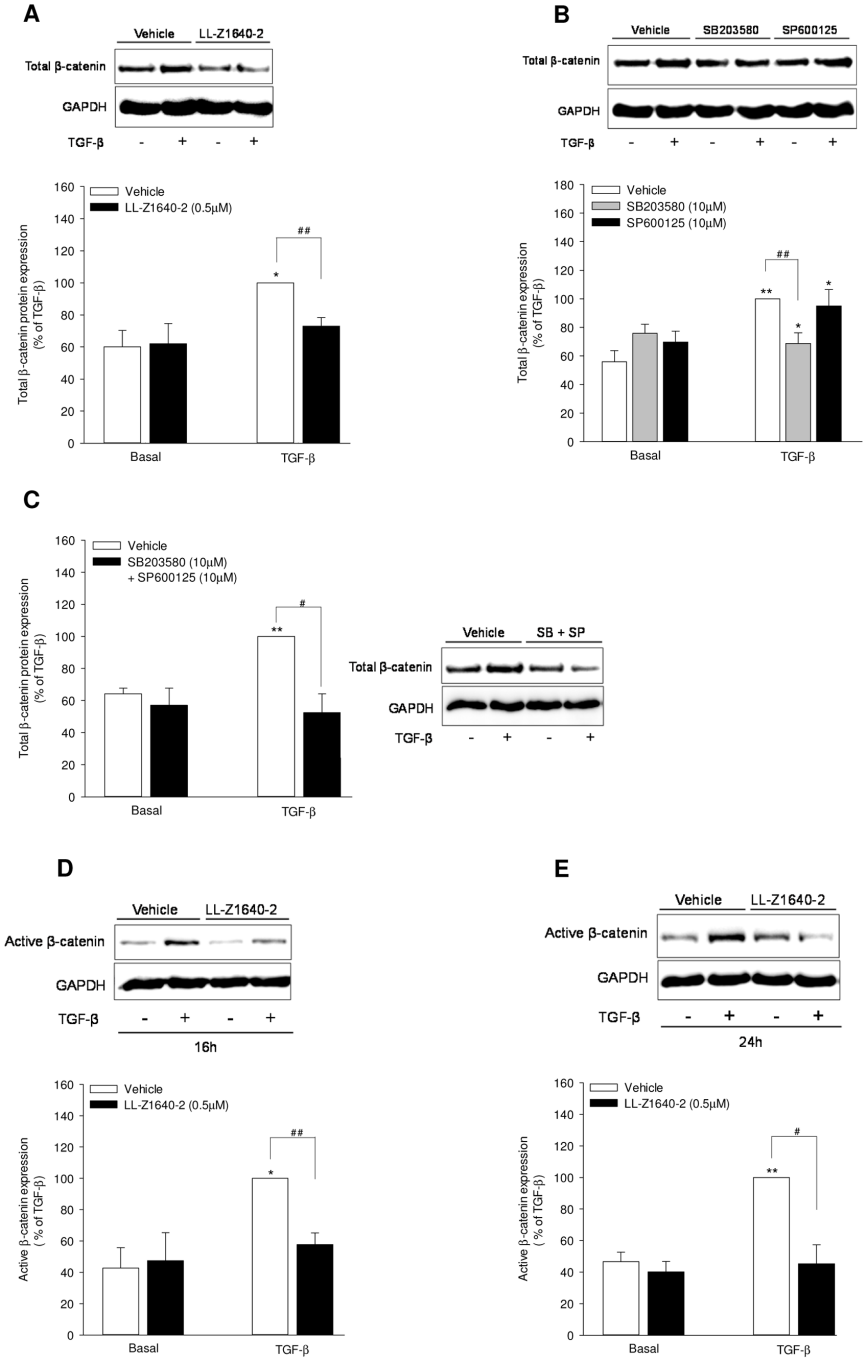
TAK1 regulates total and active fraction of β-catenin in airway smooth muscle cells. (A-C) TAK1 signaling in total β-catenin regulation. Airway smooth muscle cells were either left unstimulated (vehicle basal) or stimulated with TGF-β (2 ng/ml) in the presence or absence of LL-Z1640-2 (0.5 µM), SB203580 (10 µM), SP600125 (10 µM) or the combination of SB203580 and SP600125 (10 µM each) for 24 hours. Whole cell extracts were subjected to western analysis for detection of total β-catenin protein abundance. GAPDH expression was examined as loading control. Graphs represent quantitation of band intensities for total β-catenin corrected for GAPDH as percentage of TGF-β-induced expression. Data represent mean ± SEM of 4-6 independent experiments. *p<0.05, **p<0.01 compared to vehicle basal, # p<0.05, ## p<0.01 compared to TGF-β-stimulated cells; 2-tailed Student's *t* test for paired observations. (D, E) Regulation of active β-catenin by TAK1. Airway smooth muscle cells were either left unstimulated (vehicle basal) or stimulated with TGF-β (2 ng/ml) in the presence or absence of LL-Z1640-2 (0.5 µM) for 16 or 24 hours as indicated. Whole cells extracts were subjected to western analysis for detection of active β-catenin protein abundance. Expression of GAPDH was assessed as loading control. Graphs represent quantitation of band intensities for active β-catenin corrected for loading control as percentage of TGF-β-induced expression. Data represent mean ± SEM of 5 independent experiments. *p<0.05, **p<0.01 compared to vehicle basal, # p<0.05, ## p<0.01 compared to TGF-β-stimulated cells; 2-tailed Student's *t* test for paired observations.

Having confirmed the role of β-catenin, we got interested in the link between TAK1 and β-catenin in WNT-5A expression. As both TAK1 and β-catenin are required for WNT-5A induction, we first investigated for possible cross-regulation. To address this, we studied β-catenin stability in the presence of LL-Z1640-2. Interestingly, we observed that the TGF-β-induced increase in total β-catenin abundance was significantly suppressed in the presence of LL-Z1640-2 by ∼68% (Figure 4A).

Next, we investigated whether p38 and JNK are involved in TAK1-mediated β-catenin regulation. Of note, while JNK inhibition had no effect, inhibition of p38 significantly attenuated TGF-β-induced total β-catenin protein abundance by ∼70% in comparison to TGF-β in airway smooth muscle cells (Figure 4B). Accordingly, simultaneous inhibition of both p38 and JNK completely attenuated TGF-β-induced increase in total β-catenin levels in airway smooth muscle cells (Figure 4C).

We were intrigued by the contrasting results that while β-catenin is required for TGF-β-induced WNT-5A expression, the reduction in total β-catenin by p38 inhibition, though substantial, totally failed to affect WNT-5A transcript levels (Figure 2B and 4B). To address this issue, we focused on the functional fraction of β-catenin - the non-phosphorylated or active β-catenin. We observed that TGF-β induced non-phosphorylated active β-catenin at 16 and 24 hours which were attenuated by the TAK1 inhibitor LL-Z1640-2 at both the 16 and 24 hours by ∼74% and ∼100%, respectively (Figure 4D, E). Notably, inhibition of p38 by SB203580 failed to yield significant effect on the TGF-β-induced increase in levels of active β-catenin at both the time points studied (data not shown).

Collectively, our data suggest that TAK1 signaling mediates regulation of β-catenin via p38 and JNK.

### Sp1 is the transcription factor for WNT-5A

**Figure 5:**
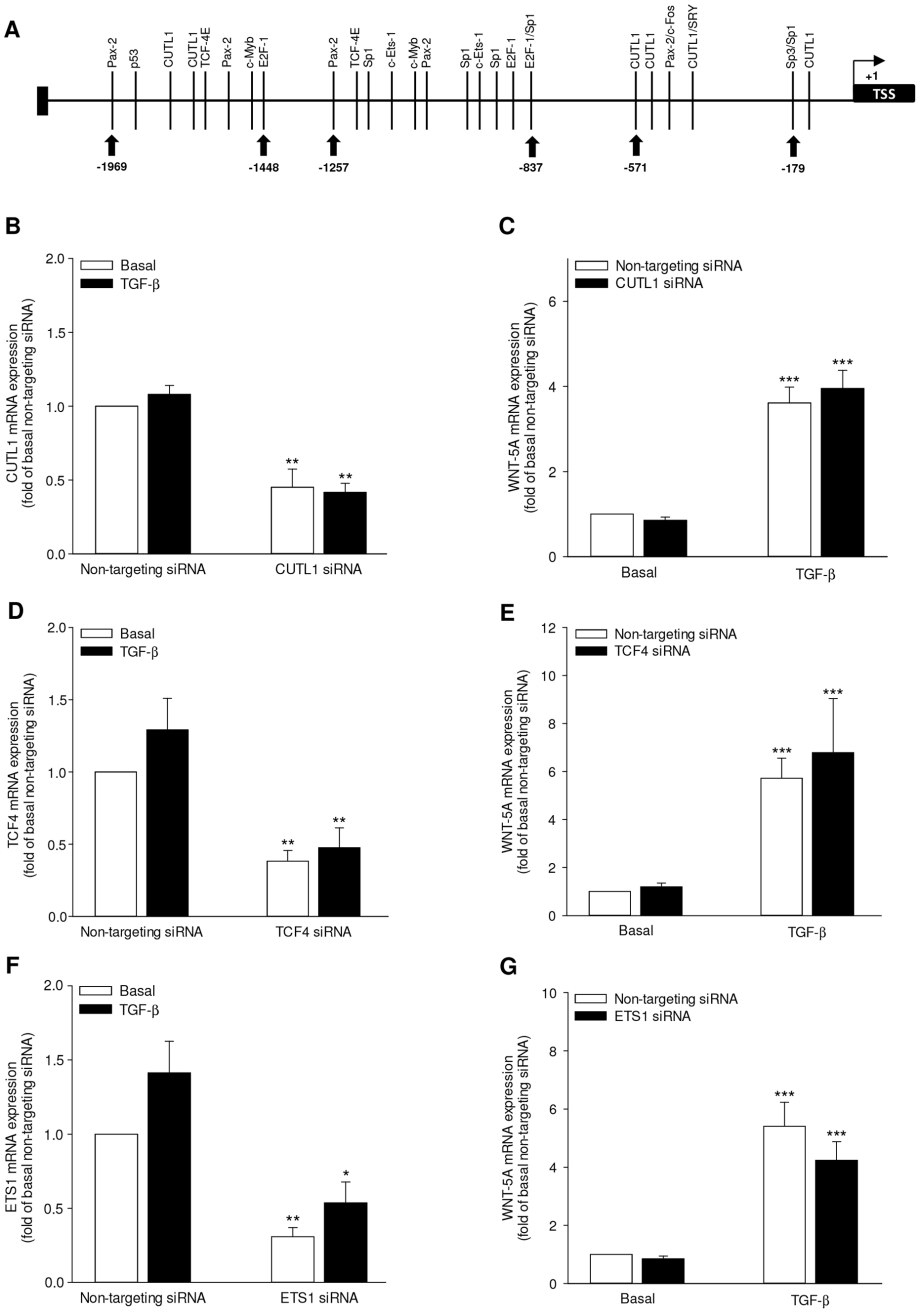
Evaluating transcriptional factors for the *WNT-5A* gene. (A) *In silico* analysis of WNT-5A promoter. Schematic representation of WNT-5A promoter A indicating the transcription factor binding sites as predicted by PROMO version 3. Only selective transcription factors are depicted here. The schematic is not to scale. TSS: Transcriptional Start Site. (B–G) Silencing of various transcription factors and WNT-5A gene expression. Airway smooth muscle cells were transfected with a non-targeting siRNA as control or with CUTL1-specific (B, C), TCF4-specific (D, E) or ETS1-specific (F, G) siRNA. Subsequently, cells were stimulated with TGF-β (2 ng/ml) for 24 hours and analyzed for the expression of genes as indicated in panels by qRT-PCR, corrected for 18S rRNA and expressed relative to non-targeting siRNA transfected, untreated control. Data represent mean ± SEM of 3-5 independent experiments. *p<0.05, **p<0.01, ***p<0.001 compared to non-targeting transfected, untreated control; 1-way ANOVA followed by Newman-Keuls multiple comparisons test.

We next sought to determine the transcription factor(s) employed by TGF-β to induce WNT-5A expression in airway smooth muscle cells. WNT-5A has two alternative promoters-A and B. To identify the potential transcription factors, we did *in silico* analysis of both the human *WNT-5A* promoter A and B as described in the Materials and Methods section which predicted binding sites for various transcription factors on both the promoters A (Figure 5A) and B (data not shown). Some of the key transcription factors and their binding sites on promoter A are presented in the diagram (Figure 5A). CUTL1 drives WNT-5A expression in pancreatic cancer cell lines whereas TCF4 is the most common transcriptional partner of β-catenin. Based on the information from the promoter analysis, our own observations from the role of β-catenin in WNT-5A induction and previous reports about WNT-5A transcriptional regulation, we targeted CUTL1, TCF4 and ETS1 using specific siRNAs. Interestingly, while specific siRNAs substantially repressed the abundance of CUTL1, TCF4 or ETS1 mRNAs confirming significant knock-down efficiency (Figure 5B, D, F), WNT-5A induction remained unaffected (Figure 5C, E, G).

**Figure 6:**
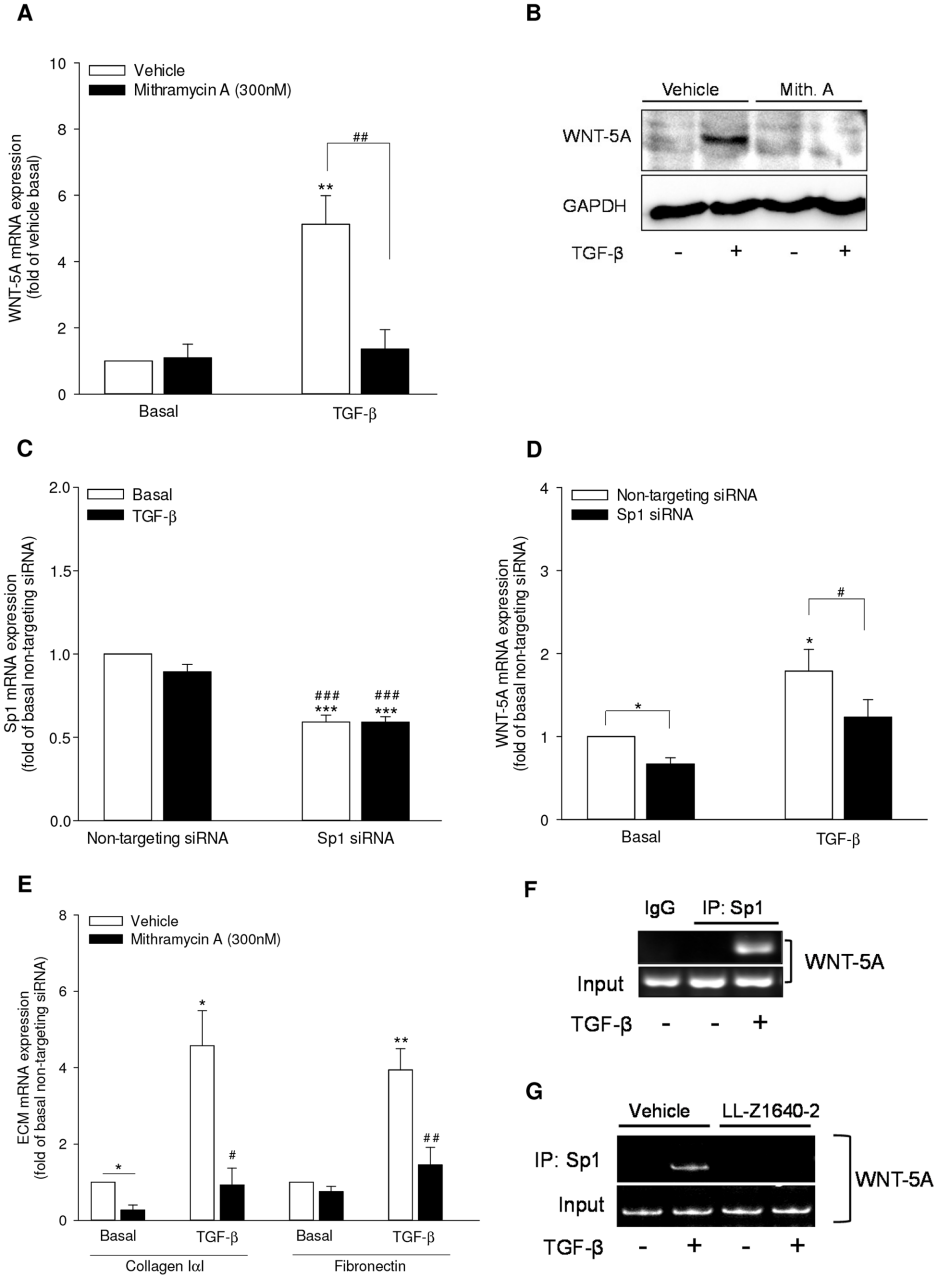
Sp1 is the transcription factor for TGF-β-induced WNT-5A expression in airway smooth muscle cells. (A-B) Mithramycin A attenuates WNT-5A mRNA and protein expression. (A) Cells were stimulated with TGF-β (2 ng/ml) in the presence or absence of Mithramycin A (300 nM) for 24 hours. WNT-5A mRNA was analyzed by qRT-PCR. Data represent mean ± SEM of 4 independent experiments. **p<0.01 compared to vehicle basal, ## p<0.01 compared to TGF-β-stimulated cells; 1-way ANOVA followed by Newman-Keuls multiple comparisons test. (B) Cells were stimulated with TGF-β (2 ng/ml) in the presence or absence of Mithramycin A (300 nM) for 48 hours. Whole cell extracts were prepared and WNT-5A protein abundance was evaluated by western analysis. GAPDH was assessed as loading control. (C, D) Cells were transfected with Sp1-specific or a non-targeting siRNA as control. Subsequently, cells were stimulated with TGF-β (2 ng/ml) for 24 hours and analyzed for the expression of Sp1 mRNA (C) and WNT-5A mRNA (D) by qRT-PCR. Data represent mean ± SEM of 5 independent experiments. *p<0.05, ***p<0.001 compared to non-targeting siRNA-transfected untreated control, #p<0.05, ### p<0.001 compared to non-targeting siRNA-transfected, TGF-β-stimulated cells; 1-way ANOVA followed by Newman-Keuls multiple comparisons test. (E) Mithramycin A attenuates TGF-β-induced extracellular matrix expression. Cells were stimulated with TGF-β (2 ng/ml) in the presence or absence of Mithramycin A (300 nM) for 24 hours. Collagen IαI and fibronectin mRNA was analyzed by qRT-PCR. Data represent mean ± SEM of 4 independent experiments. *p<0.05, **p<0.01 compared to vehicle basal, #p<0.05, ## p<0.01 compared to TGF-β-stimulated cells; 1-way ANOVA followed by Newman-Keuls multiple comparisons test. (F) Sp1 is recruited to WNT-5A promoter in response to TGF-β. Cells were left untreated or stimulated with TGF-β (2 ng/ml) for 16 hours. Chromatin was prepared and ChIP analysis was performed as described in the Materials and Methods section. PCR was carried out using primers specific for Sp1 binding region on *WNT-5A* promoter A after immunoprecipitation with anti-Sp1 or control IgG antibody. Input DNA from chromatin preparation before immunoprecipitation was amplified to ascertain the loading. Resulting PCR products were analyzed by DNA PAGE. (G) TAK1 mediates recruitment of Sp1 to *WNT-5A* promoter in response to TGF-β. Cells were left untreated or stimulated with TGF-β (2 ng/ml) in the presence or absence of LL-Z1640-2 (0.5 µM) for 16 hours. ChIP analysis was performed as described above.

Further scrutiny of *WNT-5A* promoter revealed multiple Sp1 binding sites on both the promoter A and B. To address Sp1 involvement in WNT-5A induction, we used Mithramycin A which is a selective inhibitor of recruitment of Sp family of transcription factors to the binding sites on promoter region. Interestingly, treatment with Mithramycin A (300 nM) totally abrogated TGF-β-induced expression of WNT-5A mRNA (Figure 6A). Accordingly, Mithramycin A also attenuated TGF-β-induced augmentation in WNT-5A protein abundance (Figure 6B).

To further validate the role of Sp1 in WNT-5A induction, we employed Sp1-specific siRNA. Transfection of specific siRNA significantly repressed Sp1 transcripts in both the unstimulated and TGF-β-stimulated airway smooth muscle cells in comparison to non-targeting siRNA transfected cells (Figure 6C). In agreement with the observations above using Mithramycin A, Sp1-specific siRNA significantly attenuated TGF-β-induced increase in abundance of WNT-5A transcripts confirming the requirement for Sp1 in WNT-5A induction (Figure 6D).

In line with the requirement of WNT-5A in TGF-β-induced ECM expression, we checked whether Sp1 inhibition shows similar effects. Interestingly, inhibition of Sp1 activity by Mithramycin A attenuated TGF-β-induced expression of collagen IαI and fibronectin (Figure 6E), further underlining the role of Sp1 in WNT-5A induction.

We next performed chromatin immunoprecipitation (ChIP) assay and validated the direct binding of Sp1 to WNT-5A promoters. Consistent with the role of Sp1 in WNT-5A induction as deduced from Mithramycin A and Sp1 siRNA, we confirmed binding of Sp1 on *WNT-5A* promoter A in response to TGF-β (Figure 6F). Of note, while the recruitment of Sp1 on *WNT-5A* promoter A was induced by TGF-β, Sp1 occupancy of promoter B was TGF-β independent (data not shown). In line with the role of TAK1 in WNT-5A induction, the TGF-β-induced Sp1 recruitment to *WNT-5A* promoter A was abrogated in the presence of TAK1 inhibitor LL-Z1640-2 (Figure 6G)

Our data, therefore, suggest that Sp1 is required for WNT-5A expression and is recruited to *WNT-5A* promoter via TAK1 in response to TGF-β in airway smooth muscle cells.

### TGF-β promotes β-catenin/Sp1 interaction

**Figure 7:**
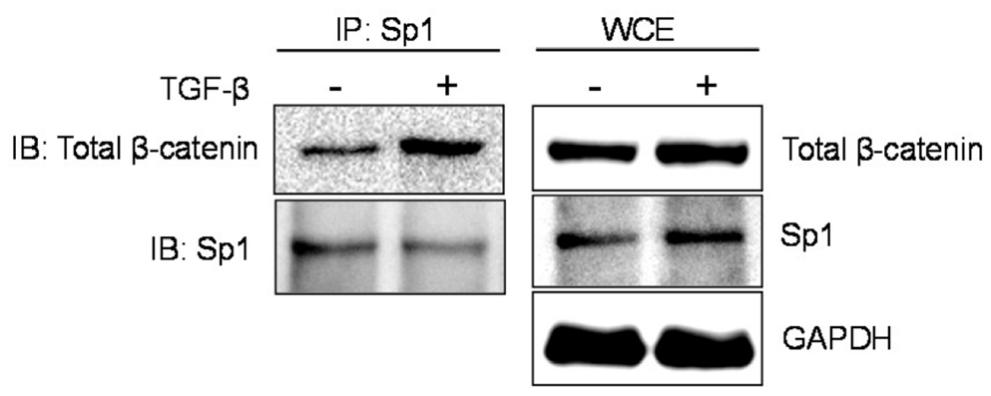
TGF-β facilitates Sp1/β-catenin interaction. Airway smooth muscle cells were stimulated with TGF-β (2 ng/ml) for 16 hours. Co-immunoprecipitation was performed as described in the Materials and Methods section. Immunocomplexes and whole cell extracts (WCE) were subjected to western analysis as indicated in the panels.

As we observed that both Sp1 and β-catenin are required for WNT-5A induction via TAK1, we sought to investigate the functional link between these findings. β-Catenin can function as transcriptional co-activator and partner with various transcription factors to regulate gene expression. We therefore determined whether β-catenin physically interacts with Sp1. Indeed, a co-immunoprecipitation assay using whole cell extracts from airway smooth muscle cells demonstrated that Sp1 associates with β-catenin (Figure 7). Interestingly, this Sp1/β-catenin interaction was further enhanced by TGF-β as indicated by increased amounts of β-catenin in Sp1 immunoprecipitates from TGF-β-stimulated cells (Figure 7). Of note, the increased interaction between Sp1 and β-catenin coincides with increased abundance of β-catenin by TGF-β as seen in whole cell extracts while Sp1 levels remain fairly equal (Figure 7).

In summary, our data demonstrate that TGF-β promotes β-catenin/Sp1 interaction.

## Discussion

In the present study, we have delineated the signaling mechanisms driving TGF-β-induced WNT-5A expression in airway smooth muscle cells. To the best of our knowledge, this is the first report describing a signaling cascade consisting of TAK1, β-catenin and Sp1 that regulates WNT-5A expression. We demonstrate that TAK1 activity is required for WNT-5A expression in response to TGF-β stimulation and provide evidence for the involvement of β-catenin in this process which, in turn, is regulated by TAK1 signaling. We further identify Sp1 as transcription factor for WNT-5A and demonstrate its interaction with β-catenin in airway smooth muscle cells. We provide evidence that Sp1 is recruited to the WNT-5A promoter in response to TGF-β, a phenomenon regulated by TAK1 activity. Collectively, our study identifies a novel pathway involved in WNT-5A regulation, thus, providing an understanding of mechanisms governing WNT-5A homeostasis.

WNT-5A plays a key role in wide range of developmental and postnatal processes and derailed WNT-5A homeostasis has been widely implicated in myriad of pathological situations [9]. WNT-5A expression is induced by a variety of growth factors and cytokines, however, little is known about the mechanisms regulating WNT-5A expression. Here, we demonstrate that TAK1 mediates WNT-5A expression in response to TGF-β as pharmacological inhibition or siRNA mediated silencing of TAK1 suppressed the TGF-β-induced augmentation in WNT-5A expression. Interestingly, out of many targeted TGF-β-activated pathways including the SMAD3-dependent cascade, only TAK1 inhibition was able to attenuate TGF-β-induced WNT-5A expression. This suggests that TAK1-mediated induction of WNT-5A is a highly selective phenomenon. TAK1 inhibition or siRNA also attenuated TGF-β induced ECM gene expression, demonstrating the functional importance of TAK1 in this response.

MAPKs including p38 and JNK are downstream effectors of TAK1 in many cell types [27]. A study from our group has shown that TAK1 mediates PDGF-induced ERK1/2 activation in airway smooth muscle cells [37]. Here, we show that TAK1 mediates TGF-β-induced activation of p38 and JNK MAPKs in airway smooth muscle cells as demonstrated by the inhibitory effect of LL-Z1640-2. We further provide evidence for direct involvement of p38 and JNK signaling in WNT-5A induction. Remarkably, only simultaneous but not separate inhibition of p38 and JNK could reduce TGF-β-induced WNT-5A expression. This clearly suggests that p38 and JNK redundantly regulate TGF-β-induced WNT-5A expression in airway smooth muscle cells.

TGF-β/SMAD constitutes the principle signaling axis in TGF-β responses [23]. We observed that the inhibition of SMAD3 enhanced TGF-β-induced WNT-5A expression, indicating a negative regulation by SMAD pathway. The contribution of TGF-β/SMAD signaling in WNT-5A induction, therefore, cannot be ruled out. Further investigation is required to decipher the regulatory role and underlying mechanisms of SMAD signaling in TGF-β-induced WNT-5A expression.

β-Catenin, the canonical WNT signaling effector, constitutes an important component in TGF-β signaling in airway smooth muscle cells [38]. In canonical WNT signaling, cytosolic β-catenin is continuously phosphorylated by a multi-component destruction complex comprising of GSK-3 and marked for proteasomal degradation. Inactivation of destruction complex by canonical WNT ligands rescues β-catenin, leading to its accumulation in cytosol. Free cytosolic β-catenin then translocates to the nucleus and activates gene transcription [1]. Besides canonical WNT ligand, TGF-β also stabilizes β-catenin where it participates in TGF-β-specific cellular responses [38]. Our group has previously identified important physiological and functional roles for β-catenin in airway smooth muscle cells [36], [39]–[41]. Here, we describe a novel role for β-catenin in WNT-5A induction. Silencing of β-catenin reduced TGF-β-induced WNT-5A induction. In addition to that, transient transfection of degradation resistant S33Y-β-catenin mutant in airway smooth muscle cells raised the basal WNT-5A protein abundance underlining the importance of β-catenin in WNT-5A induction. Remarkably, the presence of the canonical WNT ligand- WNT-3A also modestly augmented WNT-5A transcription, raising the possibility that β-catenin stabilization constitutes a primary phenomenon in WNT-5A expression in airway smooth muscle cells. However, WNT-3A-induced WNT-5A expression was much weaker in comparison to TGF-β-mediated induction suggesting that pathways other than stable β-catenin, define the magnitude of WNT-5A expression levels.

TGF-β engages a two pronged mechanism to increase the cytosolic abundance of β-catenin in airway smooth muscle cells- first, it inactivates GSK-3, the key upstream mediator of β-catenin degradation and second, it induces transcriptional upregulation of β-catenin [38]. Here, we demonstrate TAK1-mediated stabilization and subsequent increase in β-catenin abundance in response to TGF-β. Using LL-Z1640-2, we show that the TGF-β-induced increase in total cytosolic β-catenin levels is attenuated on TAK1 inhibition. This is in line with a recent report showing the positive effect of TAK1 on β-catenin stabilization and nuclear localization in KRAS-dependent colon cancer cells [42]. Furthermore, we extend our findings by demonstrating that TAK1 inhibition reduces transcriptionally active non-phosphorylated β-catenin, linking the TAK1-mediated regulation of β-catenin to functional level. The downstream mediators of TAK1 signaling- p38 and JNK- redundantly mediate β-catenin regulation in response to TGF-β. Interestingly, we also observed that TAK1 activity mediates TGF-β-induced GSK-3 inactivation by phosphorylation at Ser9-GSK-3α and Ser21-GSK-3β (data not shown). The observed GSK-3 phosphorylation sites are targeted by PI3K/AKT signaling [43] indicating the possible activation of PI3K/AKT by TAK1 in response to TGF-β. Indeed, TGF-β has been shown to activate AKT pathway via TAK1 signaling [44]. Multiple signaling pathways activated by TAK1 explain the redundancy we observe in TAK1 signaling with respect to WNT-5A induction. Altogether, our study identifies TAK1 as an upstream regulator of β-catenin, mediating its effects via a signaling cascade comprising of GSK-3, p38 and JNK. As PI3K inhibition failed to alter WNT-5A abundance, the relative contributions of GSK-3 and p38/JNK in β-catenin stabilization and WNT-5A expression warrant further investigation.

Altered expression patterns of WNT-5A and β-catenin have been implicated in various disorders, for instance, fibrosis. Enhanced expression and increased nuclear localization of β-catenin have been shown in idiopathic pulmonary fibrosis (IPF) [45], [46], systemic sclerosis [47] and has also been linked to liver [48] and renal fibrosis [49]. Similarly, increased WNT-5A expression levels have also been linked to lung [11], hepatic [13] and renal [12] fibrosis. Recently, two separate studies from our lab have also shown that WNT-5A and β-catenin mediate common function in TGF-β signaling in the airway smooth muscle cells [16], [39]. We have shown that TGF-β-induced WNT-5A mediates ECM production in airway smooth muscle cells [16] whereas another report shows that β-catenin is required and sufficient to induce ECM production in airway smooth muscle cells, even in the absence of TGF-β [39], [50]. Paradoxically, WNT-5A has been shown to both activate and antagonize β-catenin signaling in a receptor-specific manner [51]. We have previously demonstrated WNT-independent regulation of TGF-β-induced β-catenin, as neither silencing of WNT-5A nor inhibition of WNT ligand secretion by IWP2 could alter TGF-β-induced β-catenin abundance in airway smooth muscle cells [16]. However, our current study provides the unanticipated but functional explanation connecting β-catenin as an upstream mediator of WNT-5A induction in airway smooth muscle cells. Together with the previous studies, our data suggest a complex cell-dependent relation between β-catenin and WNT-5A.

Transcriptional upregulation of WNT-5A has been reported in several studies. The *WNT-5A* gene generates two very identical transcripts by utilization of alternative transcription start sites of which the corresponding upstream sequences are termed as promoter A and B [32], [33]. Both the promoters have comparable transcriptional potential; their activity, however, is highly context dependent. For instance, *WNT-5A* promoter A has been suggested to be more active in human and murine fibroblasts [33]. CUTL1 [17], STAT3 [52], TBX1 [53], NFκB [18], [19] have all previously been reported as transcription factors for WNT-5A in various cell types. We performed *in silico* analysis of *WNT-5A* promoters which revealed multiple putative transcription factor binding sites on both the promoters. Our *WNT-5A* promoter screen predicted previously described transcription factor binding sites underlining its accuracy. Silencing of CUTL1 or ETS1 failed to affect WNT-5A induction in airway smooth muscle cells suggesting a cell-specific transcriptional program regulating WNT-5A expression. Our observations regarding involvement of β-catenin in WNT-5A induction lead us to target TCF4, the most common binding partner of β-catenin. However, TCF4 knock-down didn't effect WNT-5A induction in our system suggesting that β-catenin does not utilize TCF4 for mediating WNT-5A induction.

Sp1, a member of Specificity protein/Kruppel-like family of transcription factors, is ubiquitously expressed and involved in regulating expression of a wide array of genes starting from early embryonic phase and extending throughout the life span [54]. TGF-β utilizes Sp1 for mediating many of its transcriptional responses [54]. Multiple putative Sp1 transcription factor binding sites on *WNT-5A* promoter have been predicted earlier [31], [33] and also appeared in our *WNT-5A* promoter screen. We used Mithramycin A and specific siRNA to deduce the role of Sp1 in WNT-5A induction. Mithramycin A is a highly selective inhibitor of Sp1 which competes for DNA binding with Sp1 and attenuates its recruitment on promoters [55]. Interestingly, pharmacological inhibition of Sp1 by Mithramycin A or Sp1 knock-down using specific-siRNA significantly attenuated TGF-β-induced WNT-5A expression confirming a vital role for Sp1 in this process. Mithramycin A also attenuated ECM gene expression in response to TGF-β, demonstrating the functional relevance of Sp1 in WNT-5A mediated responses in airway smooth muscle cells. ChIP analysis further validated the crucial role for Sp1 in WNT-5A induction where we demonstrate direct binding of Sp1 on *WNT-5A* promoter in TGF-β-dependent manner. Furthermore, we identified TAK1 as upstream regulator of TGF-β-induced recruitment of Sp1 as LL-Z1640-2 treatment reduced Sp1 binding to *WNT-5A* promoter in airway smooth muscle cells. This is in contrast with earlier reports where TAK1 has been shown to negatively regulate Sp1 activity in keratinocytes and lung adenocarcinoma cells [56], [57]. However, our data firmly supports positive interaction between Sp1 and TAK1 as inhibition of Sp1 completely abrogated WNT-5A expression, an effect which is strikingly similar to inhibition of TAK1. This ambiguity in observations underlines the context-dependent regulation of Sp1 by TAK1.

Sp1 activity is influenced by multiple post-translational modifications governing its DNA binding activity and protein stability [58]. MAPKs including p38 and JNK can regulate Sp1 via phosphorylation. A study has reported association of Sp1 with p38 in fibroblasts leading to subsequent phosphorylation and increased recruitment of Sp1 to *filamin A* promoter [59]. Similarly, LPS-activated p38 regulates Sp1 binding to human *il-10* promoter in human monocytes [60] whereas it regulates Sp1 transactivation, and not DNA binding, on *platelet-activating factor acetylhydrolase* (*PAF AH*) promoter in murine and human immune cells [61]. Likewise, JNK-mediated phosphorylation regulates Sp1 binding on human *urokinase-type plasminogen activator* (*uPA)* gene promoter [62] and regulates Sp1 protein stability during mitosis [63]. Sp1, hence, can be differentially regulated by MAPK signaling, not only in a cell- and stimulus-specific manner but also in a promoter-specific manner. Consistent with the positive regulation of Sp1 by both p38 and JNK signaling, activation of either p38 or JNK cascade is sufficient to sustain TGF-β-induced and TAK1-mediated transcriptional upregulation of WNT-5A. Our data, thus, suggest that TAK1 signaling recruits Sp1 to WNT-5A promoter via activation of p38 and JNK. Of note, this observation also provides an explanation to the stimulatory effect of TAK1 on Sp1 in our system as opposed to the inhibitory effect of TAK1 on Sp1 activity as reported by other groups.

Both β-catenin and Sp1 can associate with various transcription factors and co-activators in a cell- and stimulus-dependent manner to mediate their cellular responses. However, the interaction between β-catenin and Sp1 has been shown to be counteractive and indirect. For instance, constitutive activation of WNT/β-catenin signaling in mouse brain represses Sp1 target gene expression via upregulation of Sp5, a Sp1 repressor protein [64]. On the other hand, Sp1 antagonizes β-catenin signaling by enhancing expression of E-cadherin which sequesters β-catenin to the membrane [65]. Here, we report a previously undetected interaction between Sp1 and β-catenin in airway smooth muscle cells which is further promoted by TGF-β suggesting a positive functional role in TGF-β cellular responses. Of note, the increased Sp1/β-catenin interaction as observed in the presence of TGF-β coincides with increased cellular abundance of β-catenin. Whether the Sp1/β-catenin interaction is spontaneous and determined by the amount of cytosolic β-catenin available in the cell or is influenced by external factors like TGF-β has yet to be determined.

In airway smooth muscle cells, TAK1 mediates cell phenotype and cigarette smoke-induced inflammation. A study from our group has shown that TAK1-mediates PDGF induced activation of ERK1/2, leading to airway smooth muscle cell proliferation and reduction in contractile proteins [37]. Pera et al also identified a pro-inflammatory role for TAK1 wherein it mediates cigarette smoke-induced release of IL-8 in airway smooth muscle cells [66]. Interestingly, WNT-5A is a key player in pro-inflammatory responses in both the immune and non-immune cells. For instance, WNT-5A is induced by LPS/IFNγ in human macrophages where it mediates release of pro-inflammatory cytokines IL-8, IL-6, IL-1β and MIP-1β [20]. Similarly, WNT-5A induces macrophage activation and release of IL-8 and CXC chemokines in human monocytes [67]. Of note, WNT-5A also mediates pro-inflammatory responses in human aortic endothelial cells, a non-immune class of cells [68]. Our current findings correlating TAK1 activity and WNT-5A expression provide evidence for their close interaction to mediate pro-inflammatory reactions.

In conclusion, our present study describes a novel signaling cascade comprising of TAK1, β-catenin and Sp1 in TGF-β-induced WNT-5A expression in airway smooth muscle cells. We deduce the molecular pathway regulating WNT-5A expression which can have implications in various physiological and pathological situations involving WNT-5A. Moreover, our study also provides a mechanistic insight intertwining TAK1, β-catenin and Sp1 which, perhaps, has a much wider applicability extending to other cell- and tissue types and processes involving these factors. Our data suggest that TAK1 regulates TGF-β-induced WNT-5A expression by two simultaneous but linked mechanisms – 1] it augments expression of β-catenin which, in turn, partners with Sp1, perhaps, finalizing a transcriptional complex and 2] it promotes binding of Sp1 transcriptional complex to WNT-5A promoter thereby allowing WNT-5A transcription. Interestingly, therapeutic tools for targeting TAK1 [26] and Sp1 [58] are available whereas small molecule inhibitors for β-catenin [1] and WNT-5A [69] with therapeutic potential are fast emerging. Our study, thus, not only sheds light on the regulatory mechanisms of WNT-5A expression but also provides multiple therapeutic targets which could be utilized to devise effective treatment strategies for wide array of diseases involving this pathway.

## Supporting Information

Figure S1 Signaling cascades in TGF-β-induced WNT-5A expression. (A-E) Airway smooth muscle cells were either left unstimulated (vehicle basal) or stimulated with TGF-β (2 ng/ml) in the presence or absence of SIS3 (3 µM), Y27632 (1 µM), LY294002 (3 µM), SB216763 (10 µM) or BIM (3 µM) for 24 hours. Expression of WNT-5A mRNA was determined by qRT-PCR, corrected for 18S rRNA and expressed relative to vehicle basal. Data represent mean ± SEM of 3-8 independent experiments. *p<0.05, **p<0.01, ***p<0.001 compared to vehicle basal, # p<0.05, ## p<0.01, ### p<0.001 compared to TGF-β-stimulated cells; 1-way ANOVA followed by Newman-Keuls multiple comparisons test.
(TIF)
