# Supplementary figures and images for: TGF-β-Activated Kinase 1 (TAK1) Signaling Regulates TGF-β-Induced WNT-5A Expression in Airway Smooth Muscle Cells via Sp1 and β-Catenin
Source: PLoS One. 2014 Apr 11;9(4):e94801. doi: 10.1371/journal.pone.0094801 (PMC3984265; doi:10.1371/journal.pone.0094801)

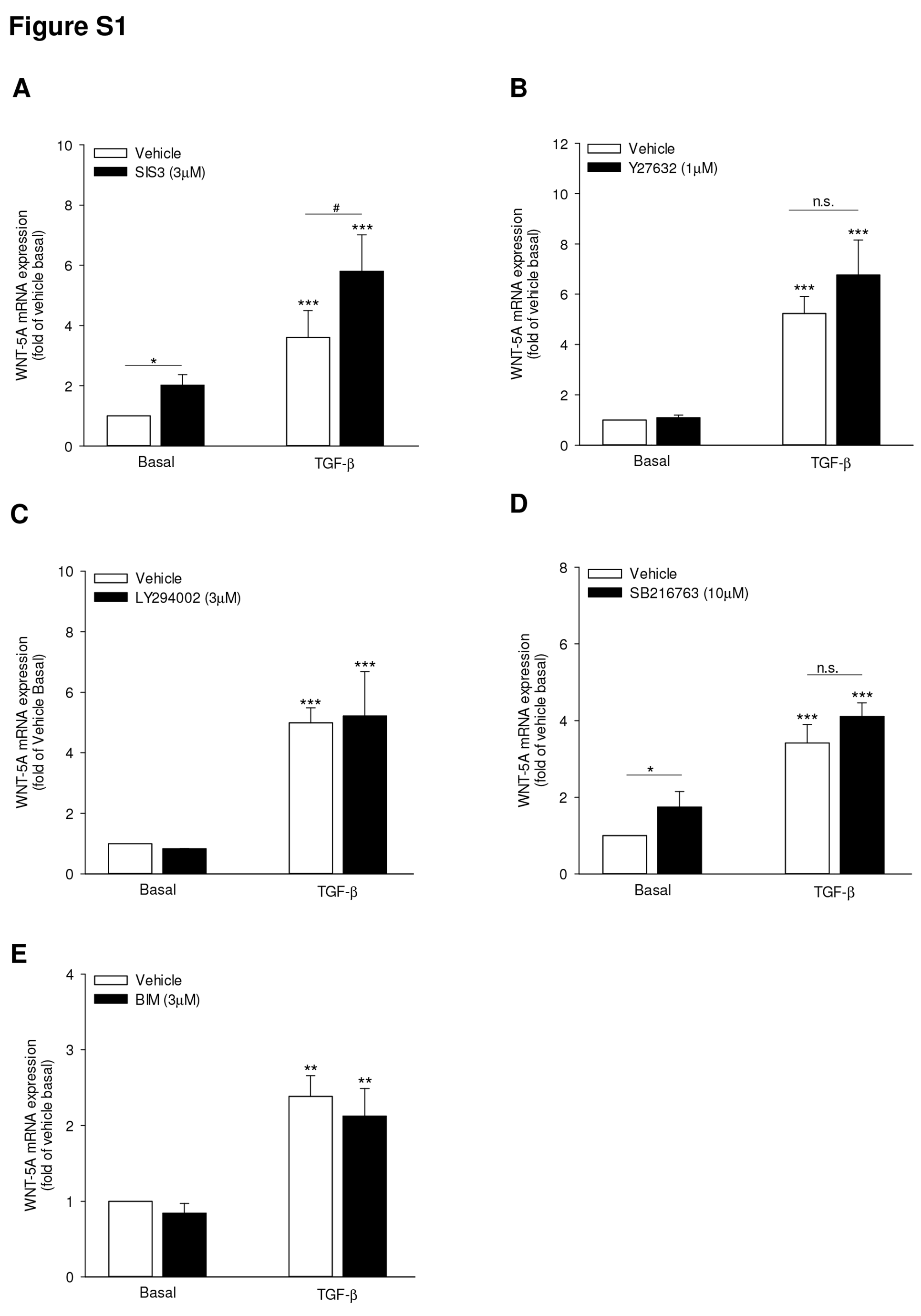

Supplement: Figure S1 — Signaling cascades in TGF-β-induced WNT-5A expression. (A-E) Airway smooth muscle cells were either left unstimulated (vehicle basal) or stimulated with TGF-β (2 ng/ml) in the presence or absence of SIS3 (3 µM), Y27632 (1 µM), LY294002 (3 µM), SB216763 (10 µM) or BIM (3 µM) for 24 hours. Expression of WNT-5A mRNA was determined by qRT-PCR, corrected for 18S rRNA and expressed relative to vehicle basal. Data represent mean ± SEM of 3-8 independent experiments. *p<0.05, **p<0.01, ***p<0.001 compared to vehicle basal, # p<0.05, ## p<0.01, ### p<0.001 compared to TGF-β-stimulated cells; 1-way ANOVA followed by Newman-Keuls multiple comparisons test. (TIF) [file pone.0094801.s001.tif]
